# High-dose vitamin D for the management acute radiation dermatitis

**DOI:** 10.1016/j.jdcr.2023.07.001

**Published:** 2023-07-08

**Authors:** Cuong V. Nguyen, Lida Zheng, Kurt Q. Lu

**Affiliations:** Department of Dermatology, Northwestern University, Feinberg School of Medicine, Chicago, Illinois

**Keywords:** radiation dermatitis, supportive oncodermatology, vitamin D

## Introduction

Radiation dermatitis (RD) is a common complication that affects patients treated with radiation therapy (RT). In the acute setting, symptoms may range from faint erythema and edema to tender cutaneous ulceration and necrosis. RD peaks 1-2 weeks following RT with therapies aimed at reducing symptoms once they occur.^1^ Unfortunately, there is no standard therapeutic regimen to alleviate the symptoms of acute RD with options limited to topical regimens including corticosteroids, herbals such as aloe vera or chamomile, and dressings that contain silver.^1^ Additional therapeutic options are needed to alleviate the symptoms of RD. In this case series, we describe a novel intervention with oral high-dose vitamin D (hdvD) and rapid symptomatic relief in 2 patients with acute RD.

## Case series

**Fig 1:**
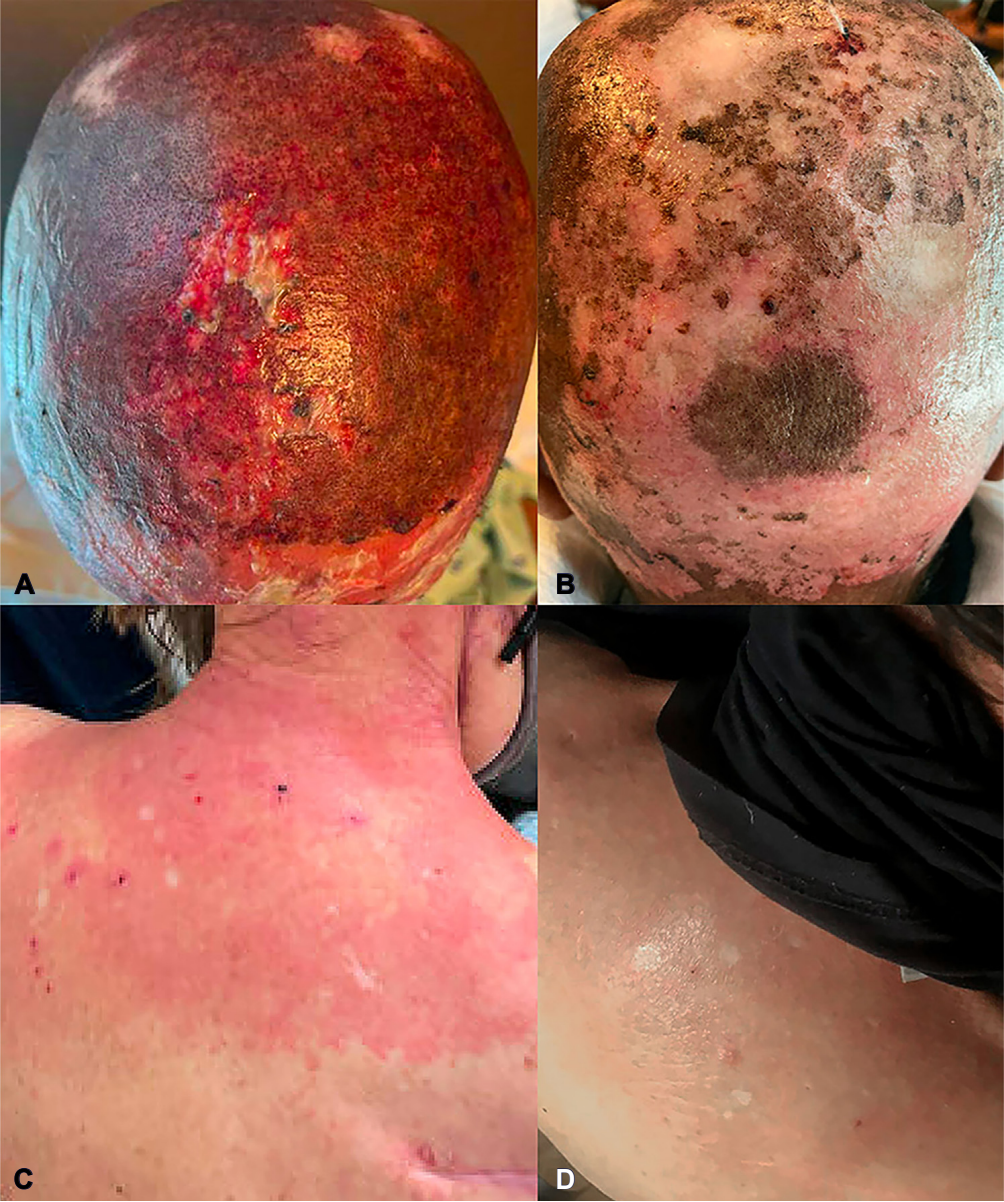
**A,** Patient 1. *Bright red* erythema with superficial ulcers prior to hdvD. **B,** Patient 1. Near complete healing of ulcers with residual *light pink* erythema and crusting 3 days post-hdvD. **C,** Patient 2. *Bright red* erythema with focal heme crusted erosions prior to hdvD. **D,** Patient 2. Residual *dull pink* erythema with *brown* post inflammatory hyperpigmentation 7 days post-hdvD.

One female patient with acute RD and 1 female patient with RD following recent fluoroscopy and CT imaging received oral hdvD (clinical features summarized in Table I). Patient 1 had previously received several gamma knife treatments for metastatic disease to the brain. She later received 37.5 Gy in 15 fractions of whole brain radiotherapy in a parallel opposed technique and 3 months later, received a course of palliative subcarinal radiotherapy. Her symptoms developed 3 days after receiving the whole brain radiotherapy without concurrent chemotherapy. Prior to radiation treatment, she had a remote history of discoid lupus erythematosus, which had minimally affected her scalp. In patient 2, RD was thought to be an acute to subacute exacerbation following fluoroscopy or a recall phenomenon in the setting of iodinated contrast, two exacerbating factors that have previously been reported in the literature.^2^^,^^3^ She had no history of autoimmune connective tissue disease. Biopsy from the back of patient 2 demonstrated diffuse fibroplasia with bizarre appearing fibroblasts, telangiectasia, and focal hyalinization highly suggestive of RD. Both patients experienced significant pain, erythema and desquamation. Patients received 100,000 IU of oral ergocalciferol once, which was repeated in a week. Topical steroids including triamcinolone 0.1% or clobetasol 0.05% ointments were also prescribed. Patients experienced improvement in pain, swelling, and redness within 3-7 days of therapy of oral hdvD. See Fig 1 for representative clinical images from before and after treatment with hdvD.

**Table I:** Clinical characteristics

Case #	Age	Sex	Underlying disease	Total radiation exposure	Time to onset from final radiation administration	Location of rash	Timing of 1st vitamin D dose after onset of rash	Time to improvement following hdvD	Baseline 25-hydroxycholecalciferol (mg/mL)	Follow-up 25-hydroxycholecalciferol (mg/mL)	Other therapies prior to hdvD	Concurrent therapies with hdvD	Additional notes
1	70’s	F	Neuroendocrine carcinoma of the pancreas	Gamma knife, whole brain radiotherapy (37.5 Gy in 15 fractions), subcarinal radiotherapy (40.05 Gy in 15 fractions)	3 d	Scalp	21 d	Within 3 d	39.5	n/a	Hydrocortisone 1% cream, Aquaphor, cephalexin, dilute vinegar soaks	Gentamicin ointment, triamcinolone 0.1% ointment	Remote history of discoid lupus erythematosus
2	60’s	F	Tonsillar carcinoma	Unknown	See notes	Back and posterior neck	7 d	Within 7 d	35.5	34.7	Hydrocortisone 1% cream, Vaseline	Clobetasol 0.05% ointment	Radiation dermatitis 1 wk after CT scan & fluoroscopy

## Discussion

Recently, the use of oral hdvD has been found to be effective in alleviating the symptoms of hospitalized patients with acute toxic erythema of chemotherapy (TEC) within 1-4 days.^4^ Similarly, administration of hdvD in patients with nitrogen mustard induced skin injury also results in rapid improvement in pain, swelling, and erythema while reducing inflammatory markers such as CCL2, CCL20, and CXCL9.^5^ While these chemokines are not associated with tumor progression, they are upregulated in the setting of inflammation. CCL2, a monocyte chemoattractant protein, is upregulated in injured tissue following RT, and its downregulation following hdvD exposure may explain the benefit of hdvD in alleviating symptoms of RD.^6^ The administration of hdvD appears to be safe. In critically ill-patients, administration of hdvD in single doses of 200,000 to 600,000 IU demonstrates a mortality benefit without negative effect on length of hospital stay or increased risk of serious adverse outcomes.^7^ An ongoing randomized control trial assessing the benefit of monthly 50,000 IU of hdvD in patients with prostate cancer is pending with smaller clinical trials having demonstrated prevention of cancer progression.^8^

In this case series, patients noted symptomatic improvement in 3-7 days following hdvD intervention, particularly in terms of pain and swelling. While it is possible that the symptoms would have resolved on their own without treatment, the degree of improvement and timing was faster than most cases of acute RD where epidermal regeneration occurs 3-5 weeks after onset with complete resolution in 1-3 months.^9^ However, while erythema did improve, the degree of improvement was not as impressive as in the case series of patients with TEC.^4^ This may be due to the deeper dermal injury associated with RT. Study limitations include the cohort size, retrospective nature, lack of long-term follow-up, and single center data acquisition. Unlike the oral hdvD study in hospitalized patients with acute TEC, the patients in this series were not followed on a day-to-day inpatient basis to assess time to response. It is possible that they also experienced symptomatic relief as soon as patients with TEC, but evaluation of this timeline was not possible. Further investigations are needed to determine optimal timing and dosing of hdvD, to determine time to clinical response, and establish the impact of therapy on cancer response.

## Conflicts of interest

None disclosed.
